# Water and nitrogen management strategies influence grain filling and yield of winter wheat in the North China Plain

**DOI:** 10.3389/fpls.2026.1795994

**Published:** 2026-03-12

**Authors:** Zhihao Cao, Guolong Gao, Yuyang Duan, Han Zhang, Chen Tian, Changxing Zhao, Xuexin Xu

**Affiliations:** Shandong Provincial Key Laboratory of Dryland Farming Technology, College of Agronomy, Qingdao Agricultural University, Qingdao, China

**Keywords:** grain filling, grain size, water–nitrogen management, winter wheat, yield formation

## Abstract

Optimizing water and nitrogen inputs during wheat’s critical developmental stages is vital for improving winter wheat yield potential and ensuring food security in the North China Plain, where water resources are increasingly constrained. A 3-year field experiment (2019–2022 winter wheat growing seasons) was conducted under a drip irrigation system to evaluate the effects of three water-saving irrigation regimes on winter wheat yield formation: DI, irrigation and split nitrogen application at jointing, booting, anthesis, and medium milk stages; *T*_J_, a single irrigation and nitrogen application at jointing; and *T*_JA_, irrigation at jointing and anthesis with a single nitrogen application at jointing. The results showed that DI significantly increased grain yield compared with *T*_JA_ and *T*_J_, primarily by enhancing thousand-grain weight and grain number per unit area without reducing spike number. The increased thousand-grain weight was associated with a higher leaf area index, enhanced antioxidant enzyme activities, reduced malondialdehyde accumulation, and sustained higher photosynthetic capacity during mid-to-late grain filling. DI also improved both the rate and duration of grain filling, particularly during the fast and slow phases, and increased grain size and favorable grain morphology (grain length, width, thickness, roundness, and seed area), thereby contributing to greater final grain weight. These findings demonstrate that coordinated water and nitrogen management at key developmental stages promotes both grain number formation and grain weight realization, providing an effective agronomic strategy to enhance wheat yield potential and resilience in areas with limited water resources.

## 1 Introduction

As an important wheat-producing region in China, the North China Plain (NCP) plays a key role in ensuring national food security. Seasonal rainfall in this region is uneven and unpredictable. Consequently, high-volume irrigation has frequently been applied in wheat production over recent decades to maximize yield (Liu et al., 2023; Bao et al., 2024). In recent years, climate change has intensified. Both the frequency and intensity of drought events have increased, thereby exacerbating regional water scarcity (Zhang et al., 2025). Additionally, given current irrigation strategies and water resource constraints, both the wheat cultivation area and the effective irrigated land in the NCP have reached their maximum thresholds (Yang et al., 2022). Developing water-saving irrigation is essential to sustain wheat production and enhance future yield potential.

Wheat yield is determined by the number of grains per unit area (the product of the number of spikes per unit area and the grains per spike) and the thousand-grain weight (Bicego et al., 2024). Since the “Green Revolution”, wheat breeders have primarily increased grain number per unit area through genetic improvements aimed at enhancing yield (Slafer et al., 2022). However, many studies have reported a negative correlation between grain number per unit area and thousand-grain weight (Beral et al., 2022; Molero et al., 2019; Vicentin et al., 2024). As the grain number per unit area increases to high levels, wheat yield approaches a plateau. Concurrently, increases in grain number are often accompanied by declines in mean grain weight, thereby constraining further yield improvement (Quintero et al., 2018; Vicentin et al., 2024). Although recent studies have focused on the physiological and genetic basis of the tradeoff between grain weight and grain number (Calderini et al., 2021; Vicentin et al., 2024), research on resolving this issue through agronomic practices remains scarce. Thus, agronomic strategies that increase grain weight without reducing grain number are critical for enhancing wheat yield potential.

The postanthesis period marks the establishment of potential grain yield, with the final actual grain yield depending on the successful realization of grain weight (Brinton and Uauy, 2019). Final grain weight relies on sufficient assimilate supply. These assimilates are derived from preanthesis reserve remobilization and postanthesis photosynthesis. The latter contributes predominantly to grain filling and final grain weight in wheat (Slafer et al., 2023). It is generally acknowledged that limited nitrogen and water supply accelerates leaf senescence, shortens the duration of green leaf area, reduces photosynthetic capacity, and decreases grain weight and yield (Yan et al., 2019). Adequate nitrogen supply is crucial for improving wheat drought tolerance. It increases leaf chlorophyll content and antioxidant capacity and also prolongs the grain-filling period, ultimately contributing to greater grain weight and yield (Ru et al., 2023; Ullah et al., 2022). Prior research has demonstrated that moderate postanthesis deficit irrigation can conserve water while mitigating leaf senescence, maintaining photosynthetic efficiency, and ensuring adequate grain filling. Grain weight is primarily governed by the grain-filling process, with strong dependence on grain-filling rate, duration, or their combined effect (Wang et al., 2024; Zhang et al., 2024). Although early anthesis and delayed leaf senescence contribute to larger grain size at maturity, the grain-filling rate is more critical than duration in determining individual grain weight (Motzo et al., 2010; Xie et al., 2016). Under abiotic stresses such as heat and drought, grain-filling rate is accelerated, but the grain-filling period is shortened (Farooq et al., 2014; Yang et al., 2004). Liu et al. (2024) found that increasing water or nitrogen application rates led to a decreasing trend in the average grain-filling rate. Therefore, optimizing the wheat grain-filling process by modulating water and nitrogen supply is a promising approach to improving grain weight and achieving yield potential.

In winter wheat production in the NCP, under conventional irrigation techniques, the optimal timing for stable yield and high water use efficiency was a single irrigation at jointing. In contrast, the optimal timing for high yield and high water use efficiency was twice irrigation at jointing and anthesis (Li et al., 2005; Xu et al., 2018). Irrigation at jointing resulted in the highest number of grains per unit area and per spike, whereas twice irrigation at jointing and anthesis increased grain yield mainly by improving thousand-grain weight (Li et al., 2005; Xu et al., 2018). Under limited irrigation conditions, can wheat grain yield be further enhanced by optimizing irrigation and nitrogen frequency to stabilize grain number and increase grain weight? To address this, irrigation and nitrogen should be applied promptly according to the critical growth stages governing winter wheat yield formation.

The objective of this study was to investigate the differences among irrigation and fertilization regimes under a drip irrigation system in terms of grain yield and yield components, flag leaf photosynthetic and antioxidant-related physiological traits, grain-filling characteristics, and grain morphological characteristics, and to clarify the mechanism by which optimized irrigation and nitrogen application frequency stabilize grain number, increase grain weight, and improve grain yield.

## 2 Materials and methods

### 2.1 Experimental site and design

**Figure 1:**
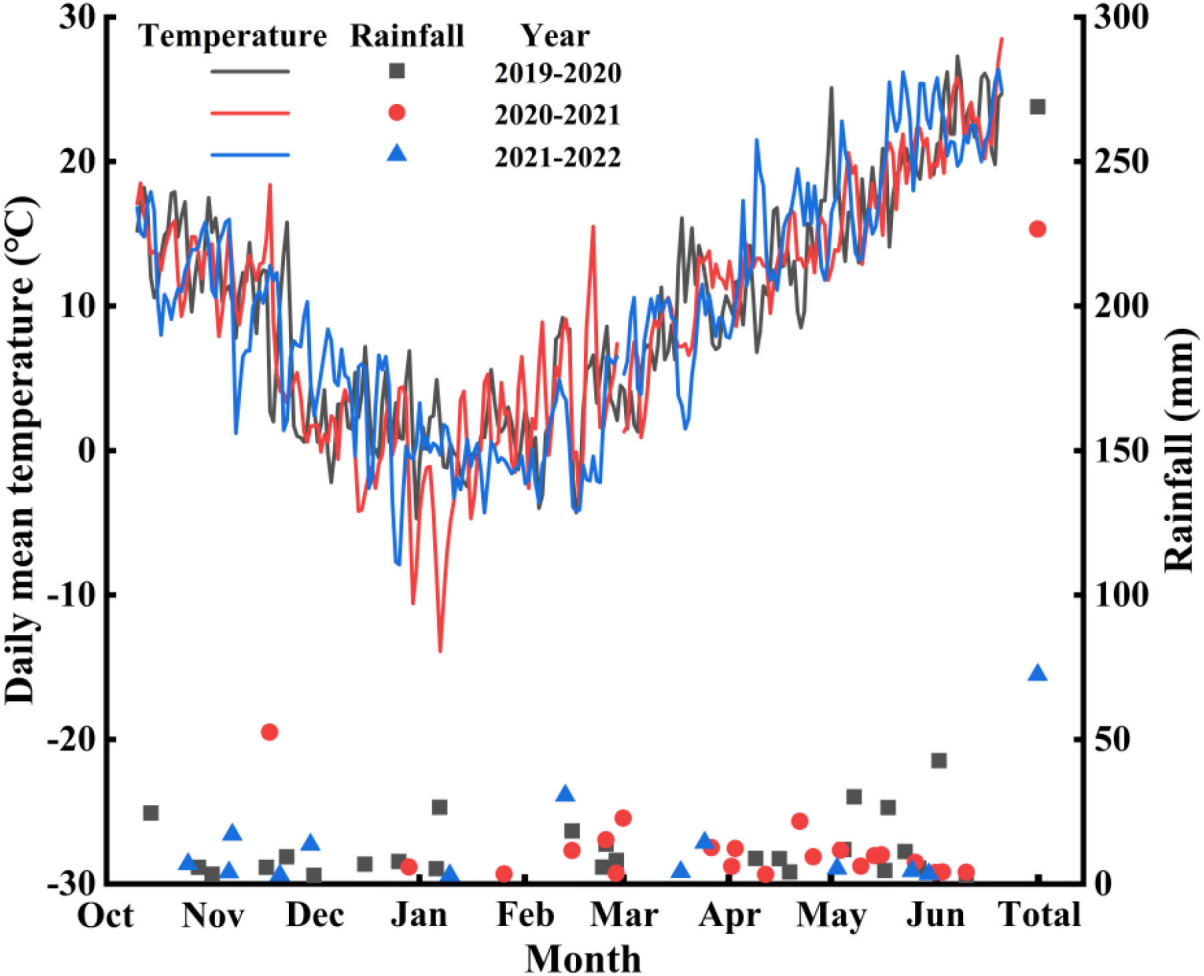
Daily mean temperature and rainfall during the 2019–2022 growing seasons.

The field experiments were performed during the winter wheat growing season in 2019−2020, 2020−2021, and 2021–2022 at Jiaozhou Modern Agricultural Science and Technology Demonstration Park of Qingdao Agricultural University, which is located on Jiaolai Street (35.53° N, 119.58° E), Jiaozhou City, Shandong Province, China. The climate type of this area is a warm temperate semi-humid continental monsoon climate. The soil at the experimental site was Shajiang black soil, classified as Vertisols according to the Chinese Soil Taxonomy and corresponding to Calcaric Vertisols under the World Reference Base for Soil Resources (WRB) system. Before the field experiment began, soil basal nutrient content at a depth of 20 cm was measured, including 0.98 g·kg^−1^ total nitrogen, 123.2 mg·kg^−1^ alkaline hydrolysis nitrogen, 28.1 mg·kg^−1^ available phosphorus, 132.8 mg·kg^−1^ available potassium, and 16.7 g·kg^−1^ organic matter. The daily mean temperature and rainfall during the winter wheat growing seasons are shown in **Figure 1**.

The Zadoks scale (Zadoks et al., 1974) was used to categorize the crop developmental stages. Three deficit irrigation regimes were established during the wheat growing seasons. The DI treatment received 30 mm of irrigation at each of the jointing (Z31), booting (Z45), anthesis (Z61), and medium milk (Z75) stages. The *T*_JA_ treatment received 60 mm of irrigation at each of the jointing and anthesis stages (Z31 and Z61), whereas the *T*_J_ treatment received a single irrigation of 60 mm at the jointing stage (Z31). The total irrigation amounts for DI, *T*_JA_, and *T*_J_ were 120, 120, and 60 mm, respectively. Detailed irrigation schedules are shown in **Table 1**. All the irrigation treatments were irrigated with drip irrigation pipes; their length and diameter were 60 m and 16 mm, respectively. The interval between the adjacent drip irrigation pipe emitters was 30 cm, and the drip irrigation pipe emitter flow rate was 2.0 L h^−1^. The plot irrigation amount was recorded using a flow meter installed at the head of each plot’s drip irrigation system. Each plot size was 60 m × 5 m, and it consisted of 24 rows of wheat spaced 20 cm apart; each treatment was replicated three times in randomized block designs. The drip irrigation pipe was laid every three rows of wheat, and the spacing between drip irrigation pipes was 60 cm.

**Table 1:** Irrigation amount and topdressing nitrogen rate of each treatment.

Treatment	Irrigation amount (mm)					Topdressing nitrogen rate (kg N ha^−1^)				
	JS (Z31)	BS (Z45)	AS (Z61)	MMS(Z75)	Total	JS (Z31)	BS (Z45)	AS (Z61)	MMS(Z75)	Total
DI	30	30	30	30	120	30	30	30	30	120
*T*_JA_	60	–	60	–	120	120	–	–	–	120
*T*_J_	60	–	–	–	60	120	–	–	–	120

Before sowing, 600 kg ha^–1^ of compound fertilizer (N–P_2_O_5_–K_2_O, 15%–15%–15%; equivalent to 90–90–90 kg ha^–1^) was incorporated as basal fertilizer. During the growing season, 120 kg N ha^–1^ was applied as topdressing, resulting in a total nitrogen input of 210 kg N ha^–1^ for all treatments. Urea used for topdressing was fully dissolved and delivered through fertigation during irrigation events. In the DI treatment, nitrogen was split into four equal applications (30 kg N ha^–1^) at each of the jointing, booting, anthesis, and medium milk stages. In contrast, the *T*_JA_ and *T*_J_ treatments received the entire 120 kg N ha^–1^ as a single application at the jointing stage. Detailed nitrogen application schedules and rates are provided in **Table 1**. The high-yielding winter wheat variety ‘Jimai22’ (*Triticum aestivum* L.) was used in this experiment. Winter wheat was sown on 14 October 2019 and 10 October 2020 at a seeding rate of 150 kg ha^–1^. During the 2021–2022 growing season, sowing was postponed to 28 October 2021 due to excessive rainfall before the optimal sowing window. Accordingly, the seeding rate was increased to 200 kg ha^–1^ in accordance with local agronomic practice to ensure adequate plant establishment under the delayed sowing conditions. Wheat plants were harvested on 16 June 2020, 19 June 2021, and 12 June 2022, respectively. Insecticides and fungicides were applied on schedule to prevent and control pests and diseases, and followed local practice during the wheat growing seasons.

### 2.2 Sampling and measurements

#### 2.2.1 Leaf area index

The green leaf area of 20 culms from each plot was measured using a Li-3000C area meter (LI-COR Inc., Lincoln, NE, USA) at anthesis (0 days after anthesis [DAA]) and at 7, 14, 21, and 28 DAA, and then the leaf area index (LAI) was calculated.

#### 2.2.2 Flag leaf gas-exchange parameters

Flag leaf gas-exchange parameters were measured at anthesis (0 DAA), 7, 14, 21, and 28 DAA. Measurements were conducted using a LI-6400 Portable Photosynthesis System (LI-COR, Lincoln, NE, USA). Measurements were performed under clear-sky conditions between 09:00 and 12:00 to minimize diurnal variation. Six biological replicates were measured per plot at each sampling time. The net photosynthetic rate (*Pn*), transpiration rate (*Tr*), stomatal conductance (*Gs*), and intercellular CO_2_ concentration (*Ci*) were recorded under a reference CO_2_ concentration of 400 μmol mol^−1^ and a photosynthetic photon flux density (PPFD) of 1200 μmol m^−2^ s^−1^ provided by an artificial light source. Leaf temperature was maintained at 25 °C ± 1°C. The flow rate was set at 500 μmol s^−1^. The leaf chamber vapor pressure deficit (VPD) averaged 1.6 kPa ± 0.1 kPa during measurements.

#### 2.2.3 Flag leaf antioxidant-related biochemical assays

Ten flag leaf samplings were randomly collected from each plot at anthesis (0 DAA) and at 7, 14, 21, and 28 DAA. Fresh samples were immediately frozen in liquid nitrogen and stored at − 80 °C until analyses.

Flag leaf tissue weighing 0.5 g was ground with 5 mL extraction buffer (0.2 M KH_2_PO_4_ and 0.2 M K_2_HPO_4_) at 0°C. The mixture was centrifuged at 10,000 × g for 20 min at 4°C, and the supernatant was divided into aliquots for enzyme analyses (Guo et al., 2015).

Superoxide dismutase (SOD) activity was assayed by measuring inhibition of the photoreduction of nitro blue tetrazolium (NBT) following the method described by Wang et al. (2013). A 20-μL portion of supernatant was transferred into a test tube and mixed with 3 mL SOD reaction liquid (0.5 M potassium phosphate buffer solution, 130 mM methionine, 750 μM NBT, 100 μM EDTA–Na_2_, and 20 μM riboflavin). The reaction mixture was exposed to 4,000 lx light for 30 min. Absorbance was determined at 560 nm using a spectrophotometer (Cary60, Agilent Technologies, Santa Clara, CA, USA). One unit of SOD activity was defined as the amount of enzyme required to inhibit NBT photoreduction by 50%.

Catalase (CAT) activity was assayed by monitoring the initial rate of H_2_O_2_ decomposition following Tan et al. (2008). A 50-μL portion of the supernatant was transferred to a test tube and mixed with 2.5 mL CAT reaction liquid (0.1 M H_2_O_2_ and 0.1 M potassium phosphate buffer with pH 7.0). Absorbance was measured thrice at 240 nm at 1-min intervals using a spectrophotometer.

Malondialdehyde (MDA) concentration was determined according to Quan et al. (2004). Flag leaves (0.2 g) were homogenized in 5 mL of 10% trichloroacetic acid (TCA) and centrifuged at 12,000 × *g* for 10 min. Next, 4.0 mL of 0.6% thiobarbituric acid (TBA) in 10% TCA was added to 2 mL of the supernatant. The mixture was heated in boiling water for 15 min, and then quickly cooled in an ice bath. After centrifugation at 12,000 × *g* for 10 min, absorbance was determined at 450, 532, and 600 nm using a spectrophotometer. MDA concentration was expressed as nanomoles per gram of fresh weight (FW).

#### 2.2.4 Grain-filling traits

Wheat spikes were marked at the beginning of the anthesis stage (Z61) on the same day. Ten marked spikes were sampled from each plot at 7-day intervals from 7 days after anthesis to 35 days after anthesis, and all spike samples were oven-dried at 105 °C for 10 min and at 70 °C until they reached a constant weight. Grains were separated from the spikes, the total number of grains was determined, and grain weight was recorded. Richards’ equation was used to fit the grain-filling process (Wang et al., 2024):


$$GW=A/{\left(1+Be^{-Kt}\right)}^{1/N}$$

Where GW is the grain weight during grain filling (mg grain^−1^), *A* is the ultimate growth quantity (mg grain^−1^), *B* is the initial value parameter, *K* is the growth rate parameter, *N* is the equation’s shaping parameter, and *t* is the days after anthesis (day).

Grain-filling trait parameters were calculated as follows:


$$V_{mean}=AK/2(N+2)$$

D=2(N+2)/K

Where *V*_mean_ is the mean grain-filling rate (mg grain^−1^ day^−1^), and *D* is the active grain-filling date (day).

The grain-filling process can be divided into three phases: gradual growth, fast growth, and slow growth, with two inflection points, denoted as *T*_1_ (day) and *T*_2_ (day). In addition, it is generally assumed that 99% of the ultimate growth is the actual end period of grain filling, then the final time is denoted as *T*_0.99_ (day), and the formula is as follows:


$$t_{1}=-ln(\frac{N^{2}+3N+N\sqrt{N^{2}+6N+5}}{2B})/K$$

$$t_{2}=-ln(\frac{N^{2}+3N-N\sqrt{N^{2}+6N+5}}{2B})/K$$

$$T_{0.99}=-ln(\frac{{\left(\frac{100}{99}\right)}^{N}-1}{B})/K$$

Accordingly, the corresponding final grain weight at the end of the gradual growth phase, the fast growth phase, and the slow growth phase are GW_1_ (mg grain^−1^), GW_2_ (mg grain^−1^), and GW_3_ (mg grain^−1^); the duration and grain-filling rate of the gradual growth phase, the fast growth phase, and the slow growth phase could be calculated as follows:


$$T_{1}=t_{1}\;V_{1}=\frac{GW_{1}}{T_{1}}$$

$$T_{2}=t_{2}-t_{1}\;V_{2}=\frac{GW2-GW_{1}}{T_{2}}$$

$$T_{3}=T_{0.99}-t_{2}\;V_{3}=\frac{GW_{3}-GW_{2}}{T_{3}}$$

Where *T*_1_ (day), *T*_2_ (day), and *T*_3_ (day) are the duration of the gradual growth phase, the fast growth phase, and the slow growth phase, respectively; and *V*_1_ (mg grain^−1^ day^−1^), *V*_2_ (mg grain^−1^ day^−1^), and *V*_3_ (mg grain^−1^ day^−1^) are the grain-filling rate of the gradual growth phase, the fast growth phase, and the slow growth phase, respectively.

#### 2.2.5 Grain morphological characteristics at maturity

Grain morphological characteristics at maturity (grain length, grain width, grain thickness, aspect ratio, roundness, and average grain area) were determined using the SeedCount SC6000R–Reflectance Image Analysis System (Next Instruments, NSW, Australia) equipped with digital image analysis software. Approximately 800–1,000 grains were evenly distributed in the SeedCount tray for three-dimensional measurements. Only one grain was placed in each groove of the tray. Each treatment included three biological replicates. Aspect ratio and roundness were autonomously calculated by the system as follows:

Aspect ratio=Grain length/Grain width.


Roundness=(Grain width/Grain length+Grain thickness/Grain length+Grain thickness/Grain width)/3

#### 2.2.6 Grain yield

Before harvest, the spike number and grain number per spike were determined from each plot. Grain yield was determined from the corresponding plot area at maturity. The 1,000-grain weight was calculated by weighing 1,000 seeds from a yield determination sample with three replicates. Grain yield and 1,000-grain weight were standardized to a 13% moisture basis.

### 2.3 Data analysis

Treatment means within each year were compared using the least significant difference (LSD) test at *p* < 0.05. A combined analysis of variance (ANOVA) was performed to evaluate the effects of year and irrigation regime, and their interaction, using SPSS 20.0 (SPSS Inc., Chicago, IL, USA). Pearson’s correlation analysis was conducted using the correlation procedure in SPSS 20.0. The grain-filling process was fitted to Richards’ equation using OriginPro 2021 R (OriginLab Corporation, Northampton, MA, USA), and all figures were generated with OriginPro 2021.

## 3 Results

### 3.1 The grain yield, yield components, and grain number

As shown in **Table 2**, all the traits were significantly affected by year (*Y*; *p* < 0.001); irrigation (*I*) also had a significant effect on most traits, except for spike number, while none of the traits were significantly influenced by year × irrigation interaction (*Y* × *I*). Across the 2019–2022 growing seasons, DI achieved the highest grain yield and thousand-grain weight, followed by *T*_JA_, whereas *T*_J_ showed the lowest values. There was no significant difference in spike number among DI, *T*_JA_, and *T*_J_. The grain number per spike in DI was significantly higher than that in *T*_J_, while no significant differences were observed between DI and *T*_JA_ or between *T*_JA_ and *T*_J_. The highest grain number per square meter was observed in DI. During the 2019–2020 growing season, grain number per square meter in DI was significantly higher than that in *T*_JA_ and *T*_J_. However, no significant difference in grain number per square meter was observed between DI and *T*_JA_ or between *T*_JA_ and *T*_J_ during the 2020–2022 growing seasons.

**Table 2:** The grain yield, yield components, and grain number of winter wheat during the 2019–2022 growing seasons.

Treatment	Spike number (10^4^ spike ha^−1^)	Grain number per spike (grain spike^−1^)	Grain number per m^2^ (10^3^ grain m^-2^)	Thousand-grain weight (g)	Grain yield (kg ha^−1^)
2019–2020					
DI	669.4 a	31.3 a	20.9 a	45.4 a	8,037.3 a
*T*_JA_	661.4 a	30.6 ab	20.2 b	42.8 b	7,302.9 b
*T*_J_	658.3 a	30.2 b	19.8 b	39.6 c	6,634.4 c
2020–2021					
DI	703.9 a	33.3 a	23.4 a	49.1 a	9,712.8 a
*T*_JA_	698.3 a	32.7 ab	22.9 ab	47.0 b	9,088.4 b
*T*_J_	691.1 a	32.4 b	22.4 b	44.3 c	8,375.2 c
2021–2022					
DI	704.1 a	34.0 a	24.0 a	48.4 a	9,814.2 a
*T*_JA_	702.3 a	33.5 ab	23.5 ab	46.1 b	9,159.5 b
*T*_J_	695.4 a	32.8 b	22.8 b	43.2 c	8,325.9 c
Mean					
DI	692.5 a	32.9 a	22.8 a	47.6 a	9,188.1 a
*T*_JA_	687.3 a	32.3 b	22.2 b	45.3 b	8,516.9 b
*T*_J_	681.6 a	31.8 c	21.7 c	42.4 c	7,778.5 c
ANOVA					
*Y*	–^***^	–^***^	–^***^	–^***^	–^***^
*I*	NS	–^***^	–^***^	–^***^	–^***^
*Y* × *I*	NS	NS	NS	NS	NS

DI, irrigation of 30 mm applied at jointing, booting, anthesis, and medium milk stages, with nitrogen split into four equal applications (30 kg N ha^−1^ each) at the corresponding stages; T_JA_, irrigation of 60 mm applied at jointing and anthesis stages, with 120 kg N ha^−1^ applied once at the jointing stage; T_J_, irrigation of 60 mm applied once at the jointing stage, with 120 kg N ha^−1^ applied once at the jointing stage; Y, year; I, irrigation; Y × I, interaction between year and irrigation. Values represent treatment means. For each growing season, n = 3; for the 3-year mean, n = 9. Within the same growing season or within the 3-year mean, values followed by different lowercase letters in the same column indicate significant differences according to the LSD test at p < 0.05. NS indicates nonsignificant differences (p ≥ 0.05); ^***^p < 0.001 indicates significant differences.

### 3.2 LAI after anthesis

**Figure 2:**
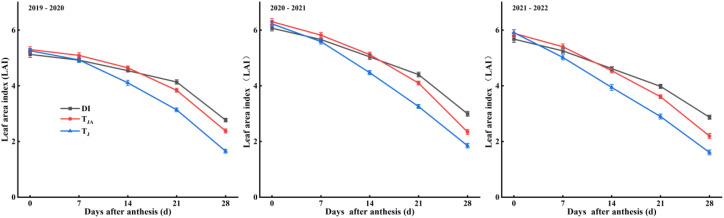
Leaf area index (LAI) after anthesis of winter wheat during the 2019–2022 growing seasons. DI, irrigation of 30 mm applied at jointing, booting, anthesis, and medium milk stages, with nitrogen split into four equal applications (30 kg N ha^−1^ each) at the corresponding stages; *T*_JA_, irrigation of 60 mm applied at jointing and anthesis stages, with 120 kg N ha^−1^ applied once at the jointing stage; *T*_J_, irrigation of 60 mm applied once at the jointing stage, with 120 kg N ha^−1^ applied once at the jointing stage. Vertical bars represent the standard errors of the means (*n* = 3).

**Figure 2** shows the LAI after anthesis across 3-year growing seasons. There was no significant difference in LAI at anthesis among DI, *T*_JA_, and *T*_J_ during the 2019–2022 growing seasons or at 7 DAA among DI, *T*_JA_, and *T*_J_ during the 2019–2021 growing season. At 7 DAA during the 2021–2022 growing seasons and at 14 DAA during the 2019–2022 growing seasons, no significant difference in LAI was observed between DI and *T*_JA_, but both were significantly higher than *T*_J_. At 21 and 28 DAA, the LAI in DI was markedly higher than that in *T*_JA_ and *T*_J_, and the LAI in *T*_JA_ was also markedly higher than that in *T*_J_.

### 3.3 Leaf antioxidant-related physiological traits

**Figure 3:**
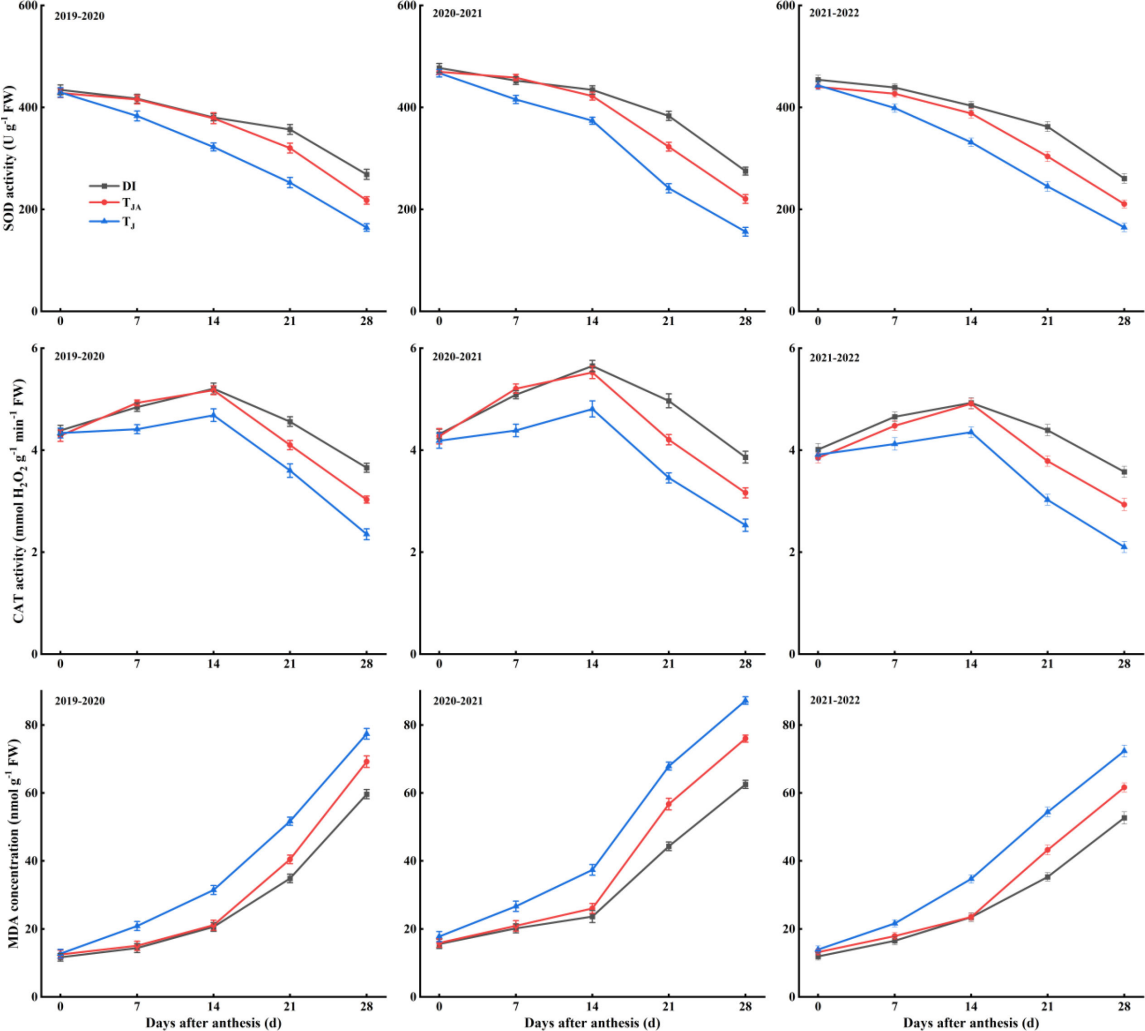
Flag leaf superoxide dismutase (SOD) activity, catalase (CAT) activity, and malondialdehyde (MDA) concentration after anthesis of winter wheat during the 2019–2022 growing seasons. DI, irrigation of 30 mm applied at jointing, booting, anthesis, and medium milk stages, with nitrogen split into four equal applications (30 kg N ha^−1^ each) at the corresponding stages; *T*_JA_, irrigation of 60 mm applied at jointing and anthesis stages, with 120 kg N ha^−1^ applied once at the jointing stage; *T*_J_, irrigation of 60 mm applied once at the jointing stage, with 120 kg N ha^−1^ applied once at the jointing stage. Vertical bars represent the standard errors of the means (*n* = 3).

The activities of SOD and CAT, as well as the MDA concentration in winter wheat flag leaves, exhibited consistent trends across the 2019–2022 growing seasons (**Figure 3**). No significant differences among DI, *T*_JA_, and *T*_J_ were observed at anthesis. After anthesis, SOD and CAT activities in DI and *T*_JA_ were significantly higher than those in *T*_J_. No significant differences between DI and *T*_JA_ were observed at 7 and 14 DAA. However, at 21 and 28 DAA, DI showed significantly higher SOD and CAT activities than *T*_JA_. In contrast, MDA concentration in DI and *T*_JA_ was significantly lower than in *T*_J_ after anthesis. Compared with *T*_JA_, DI significantly reduced MDA concentration at 21 and 28 DAA.

### 3.4 Photosynthetic characteristics

**Figure 4:**
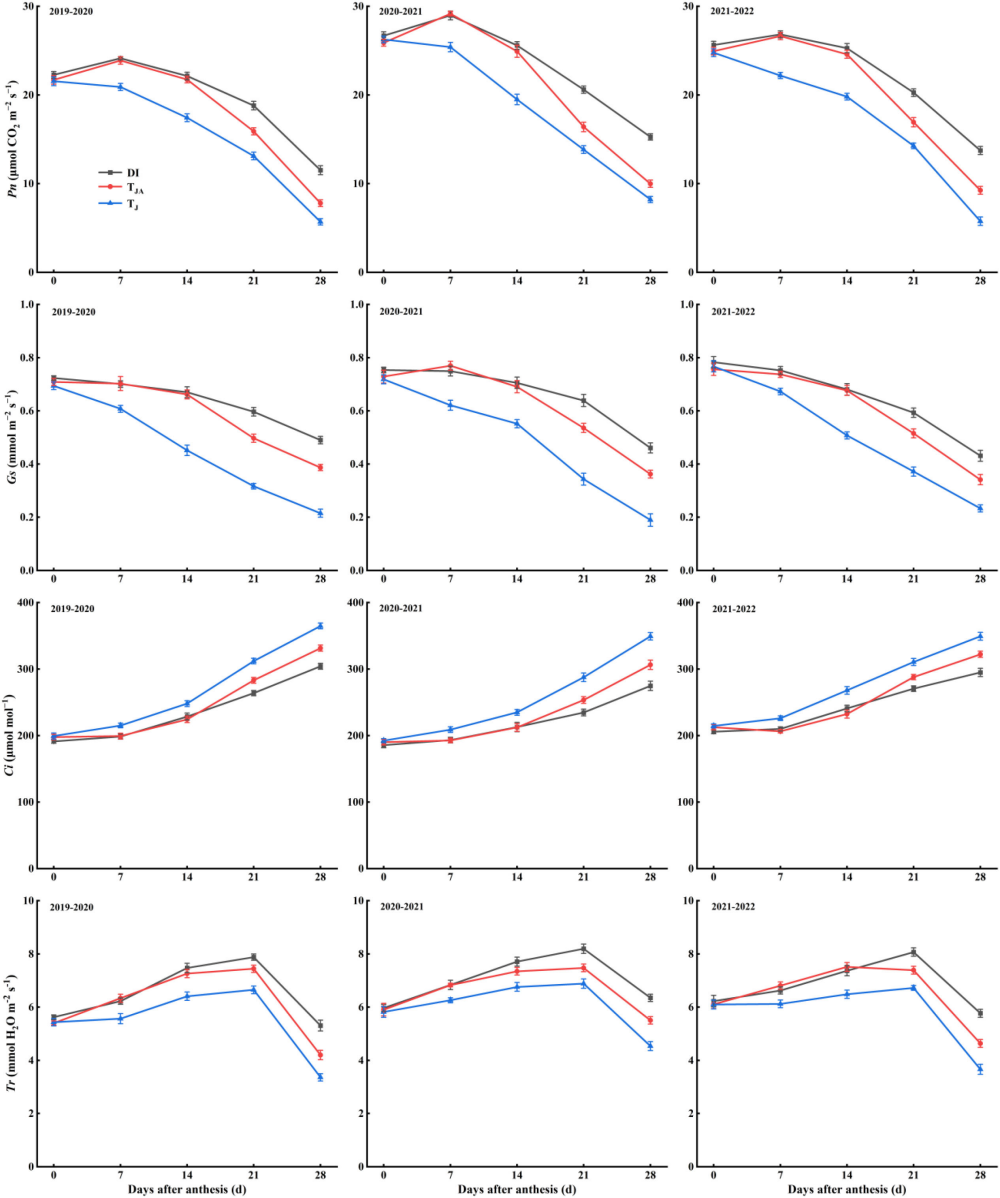
The net photosynthesis rate (*Pn*), stomatal conductance (*Gs*), intercellular CO_2_ concentration (*Ci*), and transpiration rate (*Tr*) after anthesis of winter wheat during the 2019–2022 growing seasons. DI, irrigation of 30 mm applied at jointing, booting, anthesis, and medium milk stages, with nitrogen split into four equal applications (30 kg N ha^−1^ each) at the corresponding stages; *T*_JA_, irrigation of 60 mm applied at jointing and anthesis stages, with 120 kg N ha^−1^ applied once at the jointing stage; *T*_J_, irrigation of 60 mm applied once at the jointing stage, with 120 kg N ha^−1^ applied once at the jointing stage. Vertical bars represent the standard errors of the means (*n* = 3).

The photosynthetic characteristics (*Pn*, *Gs*, *Ci*, *Tr*) after anthesis are shown in **Figure 4**. Across the 2019–2022 growing seasons, there was no significant difference in photosynthetic characteristics among DI, *T*_JA_, and *T*_J_ at anthesis. The *Pn*, *Gs*, and *Tr* in DI and *T*_JA_ were significantly higher than *T*_J_. No significant difference was observed between DI and *T*_JA_ at 7 and 14 DAA. The DI treatment exhibited significantly higher *Pn*, *Gs*, and *Tr* than the other treatments at 21 and 28 DAA. In contrast, *Ci* in DI and *T*_JA_ were significantly lower than that in *T*_J_ after anthesis, and *Ci* in DI was also lower than that in *T*_JA_ at 21 and 28 DAA.

### 3.5 Grain-filling traits

#### 3.5.1 The dynamics of thousand-grain weight

**Figure 5:**
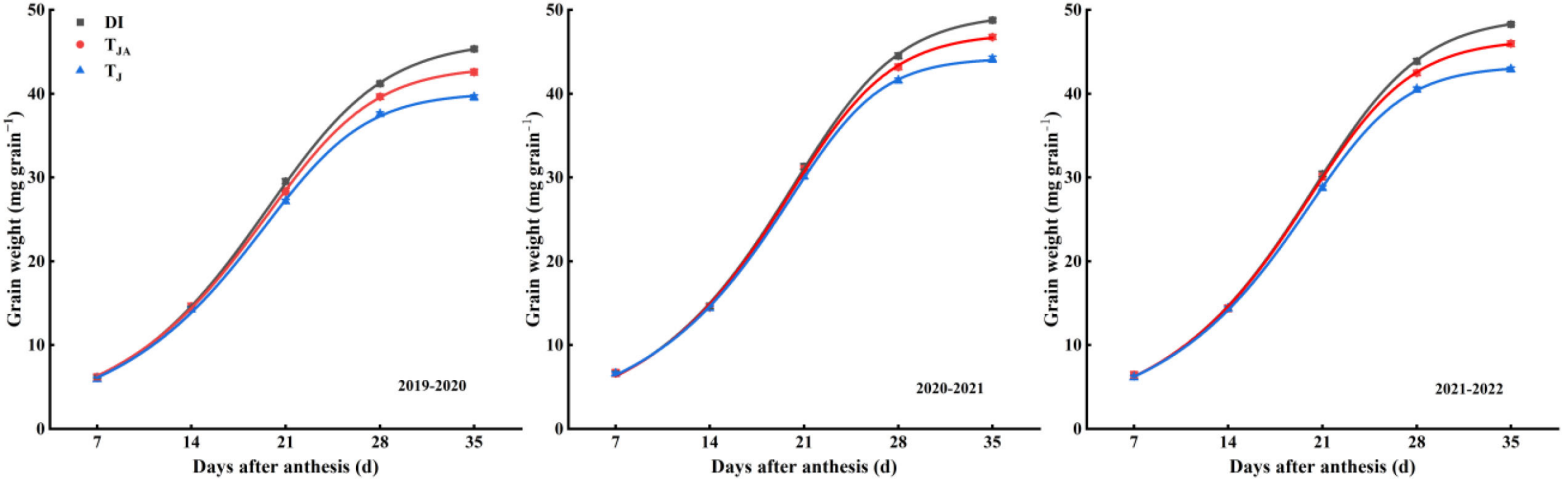
Richards’ simulation curve of grain filling in winter wheat during the 2019–2022 growing seasons. DI, irrigation of 30 mm applied at jointing, booting, anthesis, and medium milk stages, with nitrogen split into four equal applications (30 kg N ha^−1^ each) at the corresponding stages; *T*_JA_, irrigation of 60 mm applied at jointing and anthesis stages, with 120 kg N ha^−1^ applied once at the jointing stage; *T*_J_, irrigation of 60 mm applied once at the jointing stage, with 120 kg N ha^−1^ applied once at the jointing stage. Vertical bars represent the standard errors of the means (*n* = 3).

The coefficients of determination (*R*^2^) for grain filling under all treatments exceeded 0.99 (**Table 3**), indicating that the Richards’ equation provided an excellent fit to the grain-filling process across treatments. All the treatments fitting equations for grain weight dynamic change showed an *S*-shaped trend of “slow–fast–slow” (**Figure 5**). During the 2019–2020 growing season, grain weight in DI was higher than that in *T*_JA_ and *T*_J_, and grain weight in *T*_JA_ was also higher than that in *T*_J_ at 21, 28, and 35 DAA. During the 2020–2022 growing seasons, grain weight in DI and *T*_JA_ was higher than that in *T*_J_ at 21, 28, and 35 DAA. Grain weight in DI was higher than that in *T*_JA_ at 28 and 35 DAA.

#### 3.5.2 Grain-filling parameters based on the Richards’ equation

As shown in **Table 3**, the DI treatment exhibited the highest ultimate growth quantity (*A*), followed by *T*_JA_ and *T*_J_. The initial value parameter (*B*), growth rate parameter (*K*), and shape parameter (N) were ranked in the order of DI < *T*_JA_ < *T*_J_.

**Table 3:** Grain-filling parameters based on the Richards’ equation of winter wheat during the 2019–2022 growing seasons.

Treatment	A	B	K	N	R^2^
2019–2020					
DI	46.24 a	257.19 c	0.25 c	1.88 c	0.9995
*T*_JA_	43.25 b	531.80 b	0.28 b	2.26 b	0.9996
*T*_J_	40.04 c	920.24 a	0.30 a	2.52 a	0.9987
2020–2021					
DI	49.46 a	631.29 c	0.28 c	2.19 c	0.9991
*T*_JA_	47.13 b	999.25 b	0.30 b	2.40 b	0.9986
*T*_J_	44.32 c	2,382.00 a	0.34 a	2.79 a	0.9995
2021–2022					
DI	49.10 a	627.35 c	0.28 c	2.19 c	0.9996
*T*_JA_	46.44 b	964.94 b	0.30 b	2.39 b	0.9996
*T*_J_	43.26 c	2,081.22 a	0.33 a	2.76 a	0.9998

*DI*, irrigation of 30 mm applied at jointing, booting, anthesis, and medium milk stages, with nitrogen split into four equal applications (30 kg N ha^−1^ each) at the corresponding stages; *T_JA_*, irrigation of 60 mm applied at jointing and anthesis stages, with 120 kg N ha^−1^ applied once at the jointing stage; *T_J_*, irrigation of 60 mm applied once at the jointing stage, with 120 kg N ha^−1^ applied once at the jointing stage; *A*, ultimate growth quantity; *B*, initial value parameter; *K*, growth rate parameter; *N*, shape parameter; *R^2^*, equation determination coefficient. Values represent treatment means (*n* = 3). Within the same growing season, values followed by different lowercase letters in the same column indicate significant differences according to the LSD test at *p* < 0.05.

#### 3.5.3 The secondary parameters of the Richards’ equation

During the 2019*–*2022 growing seasons, the mean grain*-*filling rate (*V*_mean_) and the time to reach 99% ultimate growth quantity (*T*_0.99_) in DI were the highest, followed by *T*_JA_ and *T*_J_ (**Table 4**). The active grain-filling duration (*D*) was longest in DI and shortest in *T*_J_. Based on two inflection points between the winter wheat grain-filling rate curve, the grain-filling process can be categorized into three phases: gradual, fast, and slow growth phases. During the 2019*–*2022 growing seasons, no significant differences were observed among treatments in duration (*T*_1_) and grain-filling rate (*V*_1_) during the gradual growth phase. During the fast growth phase, DI exhibited longer duration (*T*_2_) and higher grain-filling rate (*V*_2_) than *T*_JA_ and *T*_J_. During the slow growth phase, DI showed the greatest duration (*T*_3_) and grain-filling rate (*V*_3_), followed by *T*_JA_ and *T*_J_.

**Table 4:** Richards’ equation secondary parameters for winter wheat grain filling during the 2019–2022 growing seasons.

Treatment	*V*_mean_ (mg grain^−1^ day^−1^)	*D* (day)	*T*_0.99_ (day)	Gradual growth		Fast growth		Slow growth	
				*T*_1_ (day)	*V*_1_ (mg grain^−1^ day^−1^)	*T*_2_ (day)	*V*_2_ (mg grain^−1^ day^−1^)	*T*_3_ (day)	*V*_3_ (mg grain^−1^ day^−1^)
2019–2020									
DI	1.49 a	31.02 a	38.03 a	13.51 a	1.02 a	12.32 a	2.02 a	12.20 a	0.59 a
*T*_JA_	1.41 b	30.74 a	36.25 b	13.85 a	1.02 a	11.70 b	1.93 b	10.70 b	0.57 b
*T*_J_	1.34 c	29.90 b	34.69 c	13.98 a	0.99 a	11.07 c	1.85 c	9.64 c	0.55 c
2020–2021									
DI	1.66 a	29.88 a	36.61 a	14.49 a	1.10 a	11.47 a	2.26 a	10.65 a	0.67 a
*T*_JA_	1.61 b	29.19 b	35.27 b	14.56 a	1.09 a	10.95 b	2.22 b	9.76 b	0.66 ab
*T*_J_	1.56 c	28.39 c	33.56 c	14.87 a	1.09 a	10.22 c	2.17 c	8.47 c	0.65 b
2021–2022									
DI	1.62 a	30.33 a	37.06 a	14.64 a	1.08 a	11.63 a	2.21 a	10.78 a	0.65 a
*T*_JA_	1.57 b	29.51 b	35.56 b	14.61 a	1.07 a	11.08 b	2.16 b	9.87 b	0.64 b
*T*_J_	1.50 c	28.93 b	34.05 c	14.90 a	1.05 a	10.45 c	2.08 c	8.71 c	0.62 c

*DI*, irrigation of 30 mm applied at jointing, booting, anthesis, and medium milk stages, with nitrogen split into four equal applications (30 kg N ha^−1^ each) at the corresponding stages; *T_JA_*, irrigation of 60 mm applied at jointing and anthesis stages, with 120 kg N ha^−1^ applied once at the jointing stage; *T_J_*, irrigation of 60 mm applied once at the jointing stage, with 120 kg N ha^−1^ applied once at the jointing stage. *V_mean_*, the mean grain-filling rate; *D*, the active grain-filling date; *T_0.99_*, the time to reach 99% ultimate growth quantity; *T_1_*, the duration of the gradual growth phase; *T_2_*, the duration of the fast growth phase; *T_3_*, the duration of the slow growth phase; *V_1_*, the grain-filling rate of the gradual growth phase; *V_2_*, the grain-filling rate of the fast growth phase; *V_3_*, the grain-filling rate of the slow growth phase. Values represent treatment means (*n* = 3). Within the same growing season, values followed by different lowercase letters in the same column indicate significant differences according to the LSD test at *p* < 0.05. NS indicates nonsignificant differences (*p* ≥ 0.05); ^***^p < 0.001 indicates significant differences.

### 3.6 Grains’ morphological characteristics

All the grains’ morphological characteristics, except grain length and roundness, were significantly affected by year (*Y*; *p* < 0.001). Irrigation (*I*) also exerted significant effects on these traits. However, the interaction between year and irrigation (*Y* × *I*) did not significantly affect any of the measured characteristics (**Table 5**). During the 2019–2022 growing seasons, compared to *T*_J_, DI significantly increased the grain length; no significant difference was observed between DI and *T*_JA_. Compared with *T*_JA_ and *T*_J_, DI significantly increased grain width, thickness, roundness, and average seed area; the lowest grain length, width, thickness, roundness, and average seed area were observed in *T*_J_. In contrast, the aspect ratio in DI was lower than in other treatments.

**Table 5:** Morphological characteristics of winter wheat grain during the 2019–2022 growing seasons.

Treatment	Grain length (mm)	Grain width (mm)	Grain thickness (mm)	Aspect ratio	Roundness	Average seed area (mm^2^)
2019–2020						
DI	6.49 a	3.50 a	3.27 a	1.85 c	0.658 a	17.2 a
*T*_JA_	6.41 ab	3.38 b	3.12 b	1.89 b	0.646 b	16.3 b
*T*_J_	6.29 b	3.26 c	2.96 c	1.93 a	0.631 c	15.2 c
2020–2021						
DI	6.54 a	3.65 a	3.33 a	1.79 c	0.660 a	17.9 a
*T*_JA_	6.48 a	3.52 b	3.20 b	1.84 b	0.649 b	17.0 b
*T*_J_	6.37 b	3.38 c	3.05 c	1.88 a	0.637 c	16.0 c
2021–2022						
DI	6.52 a	3.57 a	3.27 a	1.83 c	0.655 a	17.6 a
*T*_JA_	6.46 a	3.45 b	3.17 b	1.88 b	0.647 b	16.8 b
*T*_J_	6.35 b	3.31 c	3.01 c	1.92 a	0.635 c	15.8 c
Mean						
DI	6.52 a	3.58 a	3.29 a	1.82 a	0.657 a	17.5 a
*T*_JA_	6.45 b	3.45 b	3.16 b	1.87 b	0.648 b	16.7 b
*T*_J_	6.34 c	3.32 c	3.01 c	1.91 c	0.635 c	15.7 c
ANOVA						
*Y*	NS	–^***^	–^***^	–^***^	NS	–^***^
*I*	–^***^	–^***^	–^***^	–^***^	–^***^	–^***^
*Y* × *I*	NS	NS	NS	NS	NS	NS

DI, irrigation of 30 mm applied at jointing, booting, anthesis, and medium milk stages, with nitrogen split into four equal applications (30 kg N ha^−1^ each) at the corresponding stages; T_JA_, irrigation of 60 mm applied at jointing and anthesis stages, with 120 kg N ha^−1^ applied once at the jointing stage; T_J_, irrigation of 60 mm applied once at the jointing stage, with 120 kg N ha^−1^ applied once at the jointing stage; Y, year; I, irrigation; Y × I, interaction between year and irrigation. Values represent treatment means. For each growing season, n = 3; for the 3-year mean, n = 9. Within the same growing season or within the 3-year mean, values followed by different lowercase letters in the same column indicate significant differences according to the LSD test at p < 0.05. NS indicates nonsignificant differences (p ≥ 0.05); ^***^p < 0.001 indicates significant differences.

### 3.7 Correlation analysis

**Figure 6:**
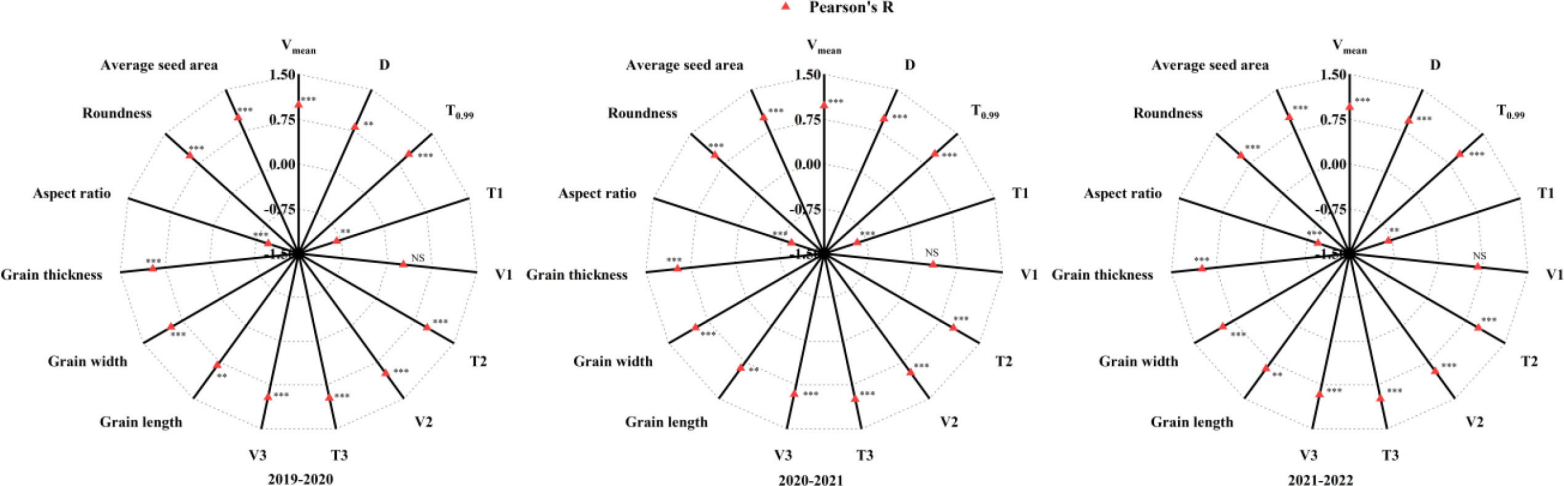
Pearson correlation coefficients of thousand-grain weight with Richards’ equation secondary parameters and grain morphological characteristics. V_mean_, the mean grain-filling rate; D, the active grain-filling date; T_0.99_, the time to reach 99% ultimate growth quantity; T_1_, the duration of the gradual growth phase; T_2_, the duration of the fast growth phase; T_3_, the duration of the slow growth phase; V_1_, the grain-filling rate of the gradual growth phase; V_2_, the grain-filling rate of the fast growth phase; V_3_, the grain-filling rate of the slow growth phase. NS, not significant (p ≥ 0.05); ^**^p < 0.01 and ^***^p < 0.001 indicate significant differences.

As shown in **Figure 6**, during the 2019*–*2022 growing seasons, thousand-grain weight was significantly and positively correlated with the mean grain-filling rate (*V*_mean_), the active grain-filling date (*D*), the time to reach 99% ultimate growth quantity (*T*_0.99_), the duration of the fast growth phase (*T*_2_), the grain-filling rate of the fast growth phase (*V*_2_), the duration of the slow growth phase (*T*_3_), the grain-filling rate of the slow growth phase (*V*_3_), the length, the width, the thickness, the roundness, and the average seed area of the grain, respectively. Thousand-grain weight was significantly and negatively correlated with the duration of the gradual growth phase (*T*_1_) and the aspect ratio, respectively.

## 4 Discussion

The period from the terminal spikelet stage to grain set is crucial for both grain number and potential grain weight formation. In contrast, the period from grain set to physiological maturity is crucial for achieving the final grain weight (Reynolds et al., 2022). Given the physiological significance of these stages in determining yield components, aligning agronomic interventions (such as irrigation and nitrogen management) with these critical periods is essential for optimizing yield outcomes (Li et al., 2021). The present results showed that DI was associated with higher thousand-grain weight and grain number per unit area compared with the other irrigation regimes (**Table 2**). The contrast between DI and *T*_J_ primarily reflects differences in total irrigation amount or split nitrogen application (**Table 1**), whereas the comparison between DI and *T*_JA_ involves differences in irrigation frequency and nitrogen application strategy. These treatment differences coincided with greater grain number per unit area and thousand-grain weight under DI, consistent with previous studies (Li et al., 2005; Zhang et al., 2025; Li et al., 2021). The observed responses may be related to improved water and nitrogen availability during the critical periods for yield component development (from terminal spikelet to grain set and from grain set to physiological maturity) (Ren et al., 2025; Xu et al., 2018; Zhang et al., 2024). Overall, the results suggest that appropriate regulation of water and nitrogen supply during key developmental stages is associated with simultaneous improvements in grain number and grain weight.

The LAI, photosynthetic capacity, and antioxidant-related physiological traits of wheat were critical physiological determinants of grain weight and yield formation. During the grain-filling stage, a high LAI and stable photosynthetic performance maintain a continuous supply of assimilates to developing grains. This supports grain weight accumulation and overall yield formation (Hamani et al., 2023). However, postanthesis drought stress may disrupt these processes by inducing membrane lipid peroxidation, leading to the accumulation of MDA and the downregulation of key antioxidant enzymes, such as CAT, peroxidase (POD), and SOD (Che et al., 2025; Lv et al., 2021). The results showed that LAI (**Figure 2**) and the activities of SOD and CAT (**Figure 3**) were higher under DI than under *T*_J_ from 7 or 14 DAA, and higher than under *T*_JA_ from 21 DAA. In contrast, MDA concentration was lower under DI than under *T*_J_ from 7 DAA, and lower than under *T*_JA_ from 21 DAA. Seasonal water input under deficit irrigation has been reported to affect leaf physiological status during grain filling (Man et al., 2017; Naseer et al., 2024). In the present study, DI received a greater total irrigation amount than *T*_J_, which coincided with higher postanthesis LAI and leaf antioxidant capacity. Previous studies have also shown that optimized irrigation scheduling or nitrogen supply is associated with the maintenance of photosynthetically active leaf area and enhanced antioxidant capacity (Wang et al., 2025; Xu et al., 2018; Yan et al., 2019). Compared with *T*_JA_, DI involved more frequent irrigation and nitrogen application, which likewise coincided with higher LAI and antioxidant capacity during the mid-to-late grain-filling period. Photosynthesis was considered an early and sensitive indicator of plant responses to environmental stress, as its capacity typically declined before other physiological functions (Lodeyro et al., 2021). The regulation of leaf *Gs* was a key physiological process in plants, as it played a crucial role in preventing desiccation while facilitating CO_2_ uptake. Under stressful conditions, the decline in photosynthetic rate was generally attributed to reduced mesophyll conductance and stomatal closure (Ashraf and Harris, 2013). In this study, *Pn*, *Gs*, and *Tr* were higher under DI than under *T*_J_ after 7 DAA, and higher than under *T*_JA_ treatment after 21 DAA. In contrast, *Ci* under DI was lower than under *T*_J_ from 7 DAA, and lower than under *T*_JA_ from 21 DAA (**Figure 4**). DI was associated with higher postanthesis photosynthetic performance compared with *T*_J_, which coincided with its higher total irrigation amount, consistent with previous reports (Naseer et al., 2024; Xue et al., 2006). Relative to *T*_JA_, the more frequent irrigation and nitrogen application implemented in DI were accompanied by higher photosynthetic performance during the mid-to-late grain-filling period, consistent with the findings of Zhao et al. (2022).

The grain-filling process mediates the translocation of photosynthetic assimilates to the storage organs. In wheat, this process exhibits distinct patterns under different nitrogen and moisture conditions (Liang et al., 2024; Wang et al., 2024; Yan et al., 2019). Wang et al. (2024) reported that grain weight was predominantly governed by the grain-filling rate, with grain-filling duration contributing only marginally. In contrast, González et al. (2014) suggested that grain-filling duration was more sensitive to environmental conditions than the rate itself. In this study, the DI treatment exhibited the highest *V*_mean_, *D*, and *T*_0.99_ (**Table 4**). Moreover, thousand-grain weight showed a significant positive correlation with *V*_mean_, *D*, and *T*_0.99_ (**Figure 6**). Hence, both the rate and duration of grain filling play critical roles in determining final grain weight, which is consistent with the study of Li et al. (2021). Grain dry matter accumulation showed an *S*-shaped trend of slow–fast–slow. According to the Richards’ equation, the grain-filling process was divided into three stages: gradual growth, fast growth, and slow growth. Xie et al. (2015) demonstrated that final grain weight was more strongly associated with the gradual and rapid phases of grain filling than with the slow phase. Consistently, other studies have highlighted that enhancing grain-filling capacity during the early and middle stages is critical for increasing grain weight (Liang et al., 2018; Wang et al., 2024). However, in our study, the grain-filling duration and rate of the fast and slow growth phases in the DI treatment were higher than those in the other treatments (**Table 4**). In addition, final grain weight was more strongly associated with the grain-filling duration and rate of fast and slow growth phases (**Figure 6**). This may be associated with the relatively high photosynthetic capacity maintained under DI during the mid- and late grain-filling stages (**Figure 4**). Grain size includes grain length, width, and thickness. It is a key agronomic trait in cereals and is positively associated with grain weight and ultimately yield (He et al., 2025). In wheat, grain size depends mainly on the rate and duration of grain filling. Grains elongate rapidly during the first ~1–5 DAA but gain little weight. Subsequently, grain width, thickness, and weight increase substantially until physiological maturity. Thus, final grain size is shaped by the early morphological phase and the subsequent active filling phase (Brinton and Uauy, 2019; Gao et al., 2024). In this study, compared with *T*_J_, both DI and *T*_JA_ increased grain length. Moreover, relative to *T*_J_ and *T*_JA_, DI significantly increased grain width, thickness, roundness, and average seed area, while reducing aspect ratio (**Table 5**). These improvements were associated with a higher grain-filling rate and a longer effective duration under DI, resulting in larger grain dimensions, consistent with previous findings (Brinton and Uauy, 2019; Geng et al., 2017). Consistently, thousand-grain weight was significantly correlated with these grain-size parameters (**Figure 6**), consistent with previous findings (Rasheed et al., 2014). These findings indicate that optimized water and nitrogen management coincided with higher grain-filling rate and duration and improved grain size, supporting greater final grain weight and yield potential.

In addition to climatic variation among seasons, a minor management adjustment occurred in the third year. The seeding rate was increased due to delayed sowing caused by excessive rainfall before the optimal sowing window. Although higher plant density may influence canopy structure and sink establishment, the two-way ANOVA showed that the interaction between year and irrigation regime (*Y* × *I*) was not significant for grain yield or yield components (*p* ≥ 0.05; **Table 2**). This indicates that the response patterns to irrigation treatments were generally consistent across seasons and were not substantially altered by the adjusted seeding rate. Therefore, this interannual management adjustment is unlikely to have affected the interpretation of treatment effects in the present study.

Furthermore, although irrigation regime and nitrogen timing were evaluated in combination, the results suggest that coordinated management during critical developmental stages was associated with improved yield component formation under water-saving conditions. Future studies that independently manipulate irrigation amount and nitrogen timing would help to disentangle their relative contributions and further clarify the underlying physiological mechanisms.

## 5 Conclusions

Under water-saving irrigation conditions, irrigation combined with split nitrogen application at the jointing, booting, anthesis, and medium milk stages (DI) was associated with higher grain number and grain weight in winter wheat, leading to increased grain yield. DI optimized water and nitrogen supply during two critical periods—terminal spikelet to grain set and grain set to physiological maturity. During the first period, DI was associated with higher grain number per spike without affecting spike number. In the second period, DI maintained a higher leaf area index and photosynthetic activity and enhanced antioxidant capacity during the mid-to-late grain-filling stage. These effects improved grain-filling rate and duration and promoted grain size development, contributing to higher final grain weight. Overall, coordinated water and nitrogen management at key developmental stages supports balanced yield component formation and provides a practical strategy for enhancing wheat productivity under limited water conditions.

## Data Availability

The raw data supporting the conclusions of this article will be made available by the authors, without undue reservation.
